# Efficacy and safety of fondaparinux in elective total hip arthroplasty and hip fracture surgery: a systematic review and meta-analysis

**DOI:** 10.1186/s13018-025-05950-6

**Published:** 2025-05-29

**Authors:** Gonzalo Mariscal, Francisco José Tarazona-Santabalbina, Oliver Marin-Peña, Erika Rotavista, Sara Arroyo Martín, María Estrella Fernández de Sevilla, Jesús Gómez-Vallejo

**Affiliations:** 1 Institute for Research on Musculoskeletal Disorders, Valencia Catholic University, Carrer de Quevedo, 2, València, 46001 Spain; 2 Geriatrics Department, University Hospital of la Ribera, Alzira, Valencia Spain; 3 Centro de Investigación Biomédica en Red Fragilidad y Envejecimiento Saludable (CIBERFES), Madrid, 28029 Spain; 4 School of Medicine, Universidad Católica de Valencia Sant Vicent Màrtir, Valencia, 46001 Spain; 5 Hospital Universitario Infanta Leonor, Madrid, Spain; 6 Hospital 12 Octubre, Madrid, Spain; 7 Medical Department, Viatris, Madrid, Spain; 8 Hospital Lozano Blesa, Zaragoza, Spain

**Keywords:** Fondaparinux, Enoxaparin, Low molecular weight heparin, Venous thromboembolism, Total hip arthroplasty, Hip fracture, Meta-analysis

## Abstract

**Background:**

As life expectancy increases, the incidence of hip fractures and the demand for total hip arthroplasties (THA) are expected to rise. This demographic shift poses significant challenges, particularly in managing post-operative complications such as venous thromboembolism (VTE), a major cause of mortality. Despite advancements, the effectiveness of various anticoagulants, in preventing VTE post-THA or hip fracture surgery remains unclear due to conflicting study results. This study aims to thoroughly evaluate the efficacy and safety of fondaparinux in patients undergoing elective THA or hip fracture surgery.

**Methods:**

This review adhered to PRISMA guidelines. Inclusion criteria targeted studies on hip surgery patients treated with fondaparinux versus placebo or other anticoagulants. Data was collected from three major databases in November 2024 using the PICOS framework, focusing on following outcomes: venous thromboembolism, mortality, and bleeding rates. Meta-analysis utilized Review Manager 5.4, and applying a fixed-effects model unless significant heterogeneity (I² ≥ 50%) was detected. Sensitivity and subgroup analyses further refined the results based on surgery type and control groups.

**Results:**

Nineteen studies (*n* = 32534) were included in the meta-analysis. Fondaparinux significantly reduced the incidence of VTE compared to controls (OR 0.43, 95% CI 0.31 to 0.61) and low molecular weight heparins (LMWHs) (OR 0.55, 95% CI 0.41 to 0.74). The incidence of distal deep vein thrombosis (DVT) was also lower in fondaparinux group compared to LMWHs (OR 0.43, 95% CI 0.31 to 0.62). Proximal DVT showed a significant reduction overall (OR 0.33, 95% CI 0.15 to 0.75) in fondaparinux group, with no significant difference compared to enoxaparin specifically (OR 0.48, 95% CI 0.20 to 1.17). Additionally, there were no substantial differences in clinically significant bleeding. The average costs (euros, pounds and/or dollars) per patient per thromboembolic event at 90 days were lower in the fondaparinux group compared to enoxaparin, both in patients undergoing elective THA (132 vs. 216) and hip fracture surgery (339 vs. 518).

**Conclusion:**

Based on the results of this meta-analysis, fondaparinux significantly reduced VTE and DVT incidence compared to LMWHs in patients undergoing elective THA and hip fracture surgery, with a similar incidence of clinically significant bleeding. Additionally, it demonstrated lower costs per thrombsoembolic event per patient than enoxaparin.

**Supplementary Information:**

The online version contains supplementary material available at 10.1186/s13018-025-05950-6.

## Introduction

Hip fractures and the need for total hip arthroplasty (THA) are on the rise in tandem with longer life expectancies and an aging population [1]. For healthcare systems around the world, this demographic transition poses serious issues, especially when it comes to managing postoperative complications [2, 3]. According to projection models, the aging population will be a major contributing factor in the huge increase in hip fracture incidence and health burden by 2029, which will present significant problems for the health and social care systems [4]. Venous thromboembolism (VTE), which includes the extremely severe conditions of deep vein thrombosis (DVT) and pulmonary embolism (PE) [5]. VTE has a major impact on quality of life, patient outcomes, and the use of medical resources [6]. VTE is one of the most common complications that lead to postoperative morbidity and mortality.

The overall mortality rate is still high even if the postoperative VTE rate is modest [5–7]. Due to their increased risk of postoperative complications and death, patients with hip fractures are especially vulnerable since they frequently have several comorbidities [5, 6]. To improve both immediate and long-term outcomes, a multidisciplinary approach might be essential [7]. During the pandemic, VTE-related death rates rose after remaining stable since 2008 [8].

Fondaparinux has shown significant potential, but its effectiveness in preventing VTE after hip fracture or THA compared with other anticoagulants remains debated. Conflicting findings from various studies have created uncertainty regarding the optimal role of fondaparinux as a prophylactic agent for VTE. Sasaki et al. demonstrated that fondaparinux had a lower incidence of VTE than enoxaparin [9]. However, other studies showed a tendency to favor fondaparinux but without statistically significant differences [10, 11]. Regarding the risk of bleeding, Erikson et al. observed less minor bleeding with enoxaparin; however, there was no difference in fatal bleeding. In contrast, Lassen et al. noted a lower incidence of DVT with fondaparinux but an increase in the number of transfusions [12]. However, the increased bleeding did not require reoperation. These inconsistent results highlight the need for a thorough analysis and careful evaluation of the available evidence.

These concerns have been the focus of previous meta-analyses, but they ran into issues that call for more research. Hur et al. [13], for instance, only examined four studies, which might not give a complete picture. Similarly, Huang et al. [14] ignored hip fractures in favor of concentrating only on total hip arthroplasty. There may be gaps in the results since these analyses did not take into consideration all the variables that were necessary to control heterogeneity.

In order to prevent VTE following THA and hip fracture surgery, this systematic review and meta-analysis sought to provide a thorough evaluation of fondaparinux’s safety and effectiveness in comparison to other options. Evidence-based recommendations for VTE prophylaxis can be influenced by these findings.

## Methods

### Eligibility criteria

The Preferred Reporting Items for Systematic Reviews and Meta-Analysis (PRISMA) were followed in the conduct of this review [15]. For this investigation, the protocol was registered with PROSPERO (CRD42025636847). To choose relevant studies, inclusion and exclusion criteria were developed. The PICOS framework was used in this study’s methodology: P (patients) comprised individuals undergoing hip fracture surgery or total hip arthroplasty; I (intervention) involved fondaparinux; C (comparator) comprised patients receiving a placebo or other therapeutic alternatives; O (outcomes) evaluated efficacy, safety and costs; and S (study design) only required comparative studies. No limitations existed on the basis of ethnicity, nation, or gender. Non-human studies (animal studies) and in vitro studies were excluded, as were certain publication types, such as book chapters, editorials, author responses, conference papers, posters, reviews, letters, patents, and papers available only as abstracts. Studies lacking extractable data, those with overlapping or unreliable data, and duplicates were also excluded.

### Information sources and search methods for identification of studies

A comprehensive search was performed across multiple databases including PubMed, Scopus, and Cochrane Library in November 2024. Specific search terms were utilized: “fondaparinux,” “hip arthroplasty,” “hip replacement”, “hip fracture”, “orthopedic” and “orthopaedic” (Additional File 1). A manual search of the references was conducted to ensure a comprehensive coverage of the literature. No language or date restrictions were applied.

The study selection process was conducted in two distinct stages: (1) title and abstract screening and (2) full-text review. Initially, all records were identified through a database search, and duplicates were removed using EndNote Software Version (X-9). Two independent reviewers screened the titles and abstracts of all the unique records to assess their potential eligibility based on predefined inclusion and exclusion criteria. Any disagreements at this stage were resolved by discussion or consultation with a third reviewer. Records deemed potentially relevant were proceeded to the full-text review stage. In this phase, the same two independent reviewers assessed full-text articles for eligibility by applying the same inclusion and exclusion criteria in greater detail. The reasons for exclusion at the full-text stage were documented. Discrepancies between the reviewers were resolved by discussion or, if necessary, by a third reviewer.

### Data extraction and data items

The data collected from these studies were independently reviewed by two authors. In cases of disagreement, a third author was consulted to resolve the issue and finalize the assessment. Baseline characteristics of the studies included region, study design, type of hip surgery, gender, age (years), BMI, history of venous thromboembolism, and doses of fondaparinux. The primary data extraction focused on VTE, defined as the number of deep vein thrombosis (DVT), pulmonary embolisms (PE), or both. Additional outcomes examined included the incidence of DVT, PE, mortality, and bleeding. For bleeding, rates of minor bleeding were recorded, along with more specific outcomes, such as bleeding in critical organs, bleeding leading to reoperation, fatal bleeding, and the number of transfusions. The costs were also analyzed. Other variables such as operative time, intraoperative blood loss, treatment duration, and creatinine levels were collected to ensure homogeneity between the groups regarding these potential confounding variables.

### Assessment of risk of bias in included studies

For non-randomized studies, the risk of bias was assessed using the Methodological Index for Non-Randomized Studies (MINORS) with 12 items [16]. For non-comparative studies, scores ranging from 0 to 4, 5–7, 8–12, and ≥ 13 were categorized as very low, low, fair, and high quality, respectively. In the comparative studies, scores ranging from 0 to 6, 7 to 10, 11 to 15, and ≥ 16 were categorized as very low, low, fair, and high quality, respectively.

The Cochrane Review Manager tool was used to assess the risk of bias in the clinical trials. Randomization, allocation concealment, participant and outcome assessor blinding, insufficient outcome data, selective outcome reporting, and other sources of bias were among the considered domains taken into consideration. Every domain was evaluated for “low risk of bias,” “uncertain risk of bias,” or “high risk of bias” [17].

### Assessment of results

Continuous variables measured on the same scale were analyzed for mean differences (MD) and 95% confidence intervals (CI). When outcomes were assessed on different scales or on the same scale with varying units, standard mean differences (SMD) with 95% CI were calculated. Odds ratios (OR) with 95% confidence intervals were used for dichotomous variables. Meta-analysis was conducted using Review Manager version 5.4.1. Heterogeneity among studies was assessed using the chi-square test and I² statistics, with I² values of 25%, 50%, and 75% indicating low, moderate, and high heterogeneity, respectively. A fixed-effects model was employed unless significant heterogeneity was detected (I² ≥ 50%). The variables from the figures were accurately extracted using WebPlot Digitizer 4.5. The handling of missing data adhered to the guidelines set forth in the Cochrane Handbook [18].

### Publication bias

Publication bias was assessed using Review Manager software, which is widely utilized for systematic reviews and meta-analyses. To visually evaluate the presence of publication bias, funnel plots were generated. Plotting the treatment effects estimated from individual trials versus a metric of study size or precision—typically the standard error—is done graphically with funnel plots. Asymmetry in the data, which may be a sign of publishing bias, can be found using this technique. The horizontal axis (x-axis) in these maps denotes the study’s size, precision, or standard error, while the vertical axis (y-axis) shows the effects of the therapy. Asymmetry may signal that smaller, possibly unpublished studies with negative or null results are not included in the analysis, whereas a symmetrical distribution of data points around the effect size shows little to no publication bias.

### Additional analysis

By removing the study with the highest weight from the comparison across all outcomes, a sensitivity analysis was carried out. For the primary outcomes, further sensitivity analyses were conducted, specifically by classifying the results according to the kind of surgery—total hip arthroplasty and hip fracture surgery.

According to the type of control groups employed, subgroup analyses were conducted using low molecular weight heparin (including studies that did not specify the type, as well as those employing enoxaparin and nadroparin), and comparisons with mechanical prophylaxis, no treatment, or placebo.

Additionally, the GRADEpro tool was used to apply the Grading of Recommendations Assessment, Development, and Evaluation (GRADE) framework [19]. This method considers variables including risk of bias, heterogeneity, precision of results, and consistency across studies in order to assess the quality of the evidence and the strength of recommendations. A thorough examination and well-informed decision-making based on the quality and dependability of the available data are guaranteed by this all-encompassing method.

## Results

### Study selection

**Fig. 1:**
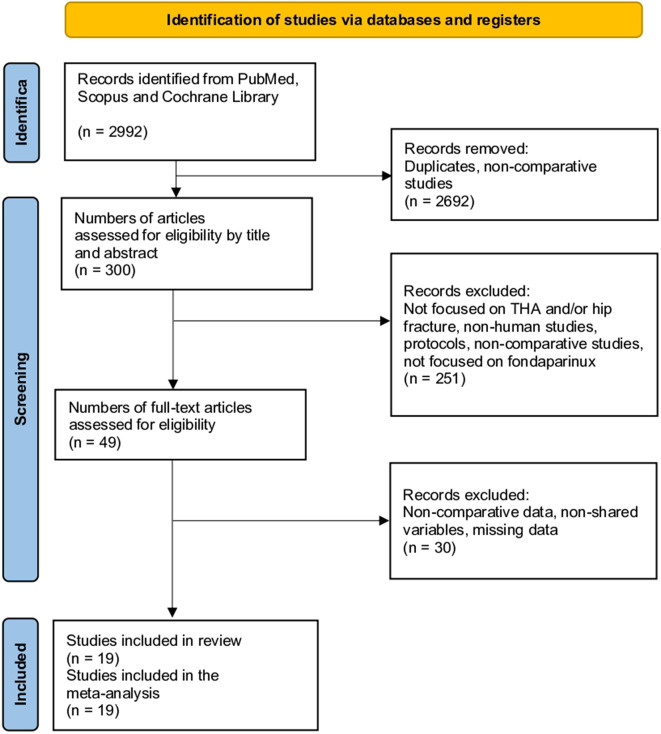
Study selection flow diagram (Preferred Reporting Items for Systematic Reviews and Meta-analyses)

The initial database search yielded 2,992 studies. After filtering to include only clinical trials and observational studies, 2,692 were excluded, leaving 300 relevant studies. A further examination of titles and abstracts resulted in the exclusion of 251 studies that did not focus on total hip arthroplasty (THA) and hip fractures, were not human studies, lacked comparative data, were merely protocols, or did not concentrate on fondaparinux. After a full-text review of the remaining 49 articles, 30 were removed due to issues such as non-comparative data, non-shared variables, and missing data. No additional articles were identified through reference checks of the included studies. Ultimately, nineteen articles met al.l criteria and were included in the meta-analysis (Fig. 1) [9–12, 20–34].

**Fig. 2:**
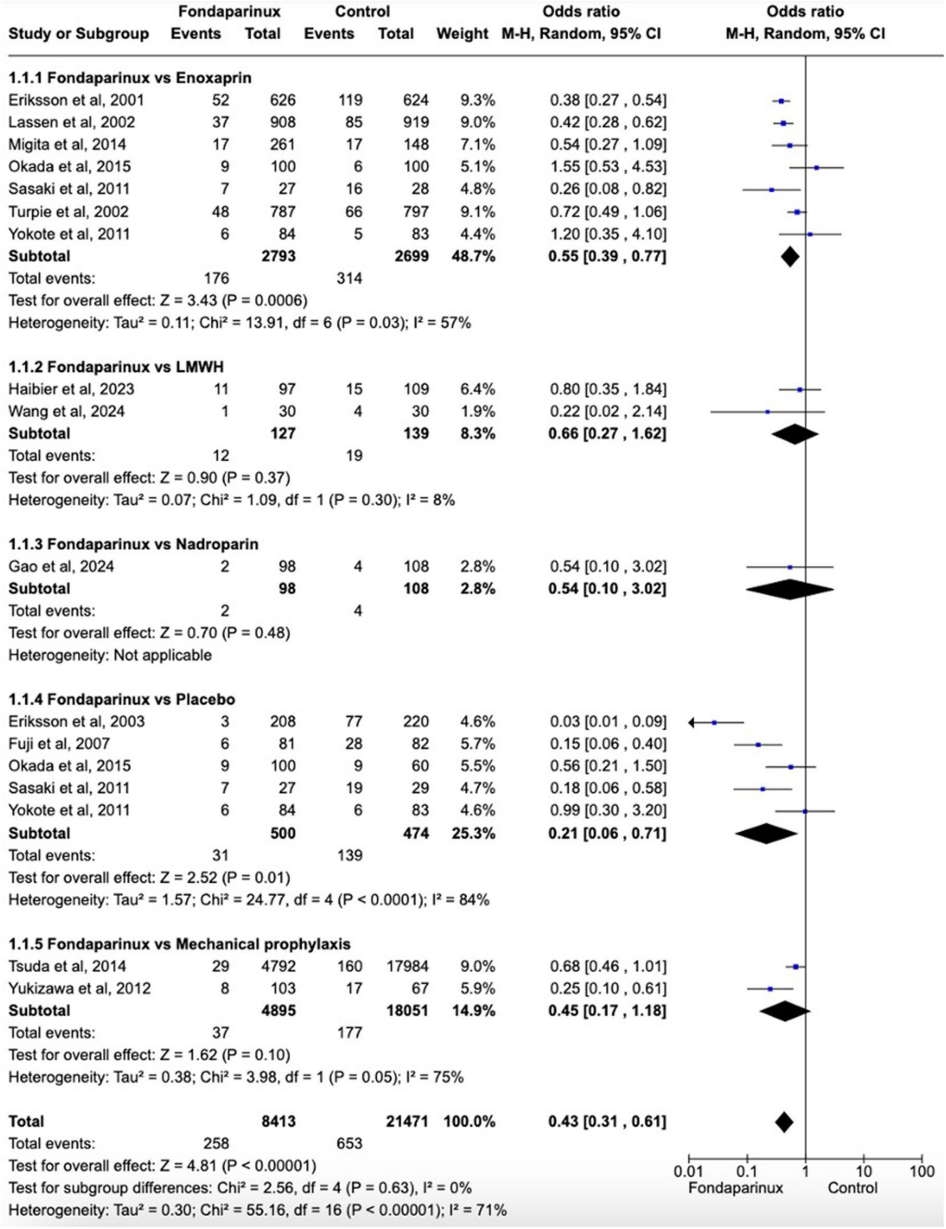
Forest plot showing effects of Fondaparinux in the incidence of VTE versus different control groups. Fondaparinux demonstrated significantly fewer VTE events compared to the control group (OR 0.43, 95% CI 0.31 to 0.61) including LMWHs (OR 0.55, 95% CI 0.41 to 0.74)

**Fig. 3:**
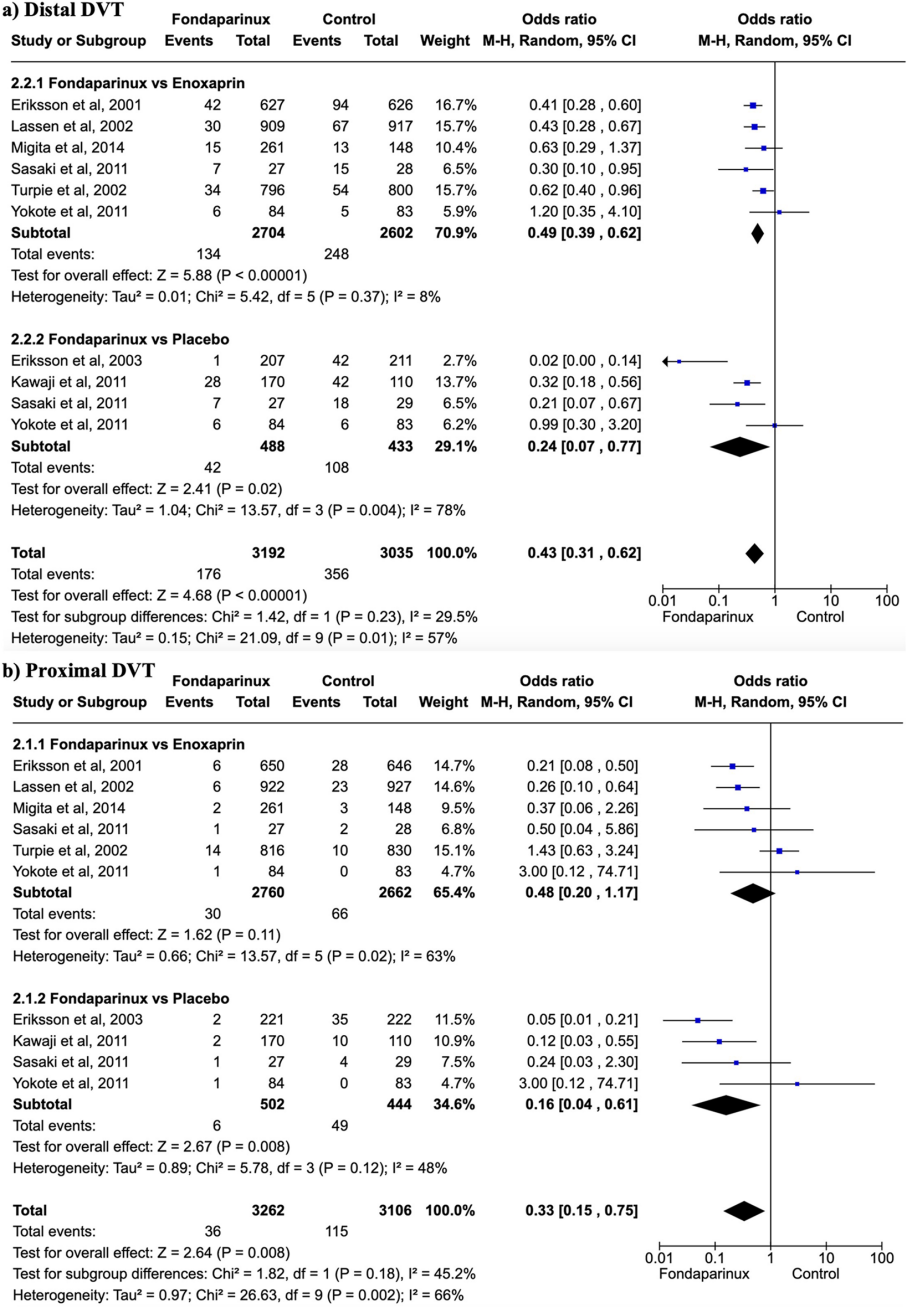
Forest plot showing effects of Fondaparinux in the incidence of distal and proximal DVT versus different control groups. (**a**) Fondaparinux showed a lower incidence of distal DVT compared to the control group (OR 0.43, 95% CI 0.31 to 0.62), including enoxaparin (OR 0.49, 95% CI 0.39 to 0.62); (**b**) fondaparinux significantly reduced the incidence compared to the control group (OR 0.33, 95% CI 0.15 to 0.75) but there were no differences compared with enoxaparin (OR 0.48, 95% CI 0.20 to 1.17)

**Fig. 4:**
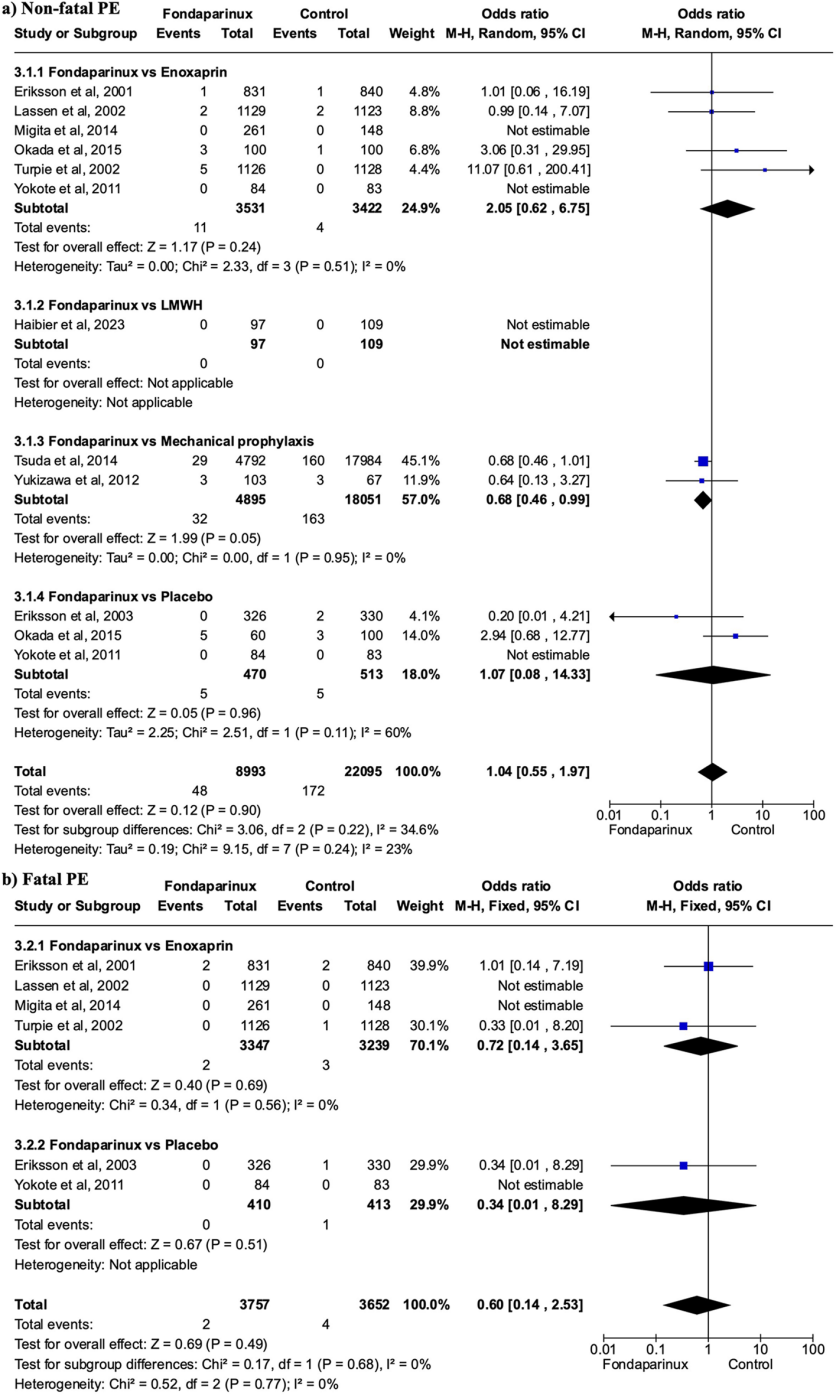
Forest Plot showing effects of Fondaparinux in the incidence of pulmonary embolism. There were no significant differences neither in the case of non-fatal pulmonary embolism (**a**) nor fatal pulmonary embolism (**b**)

**Fig. 5:**
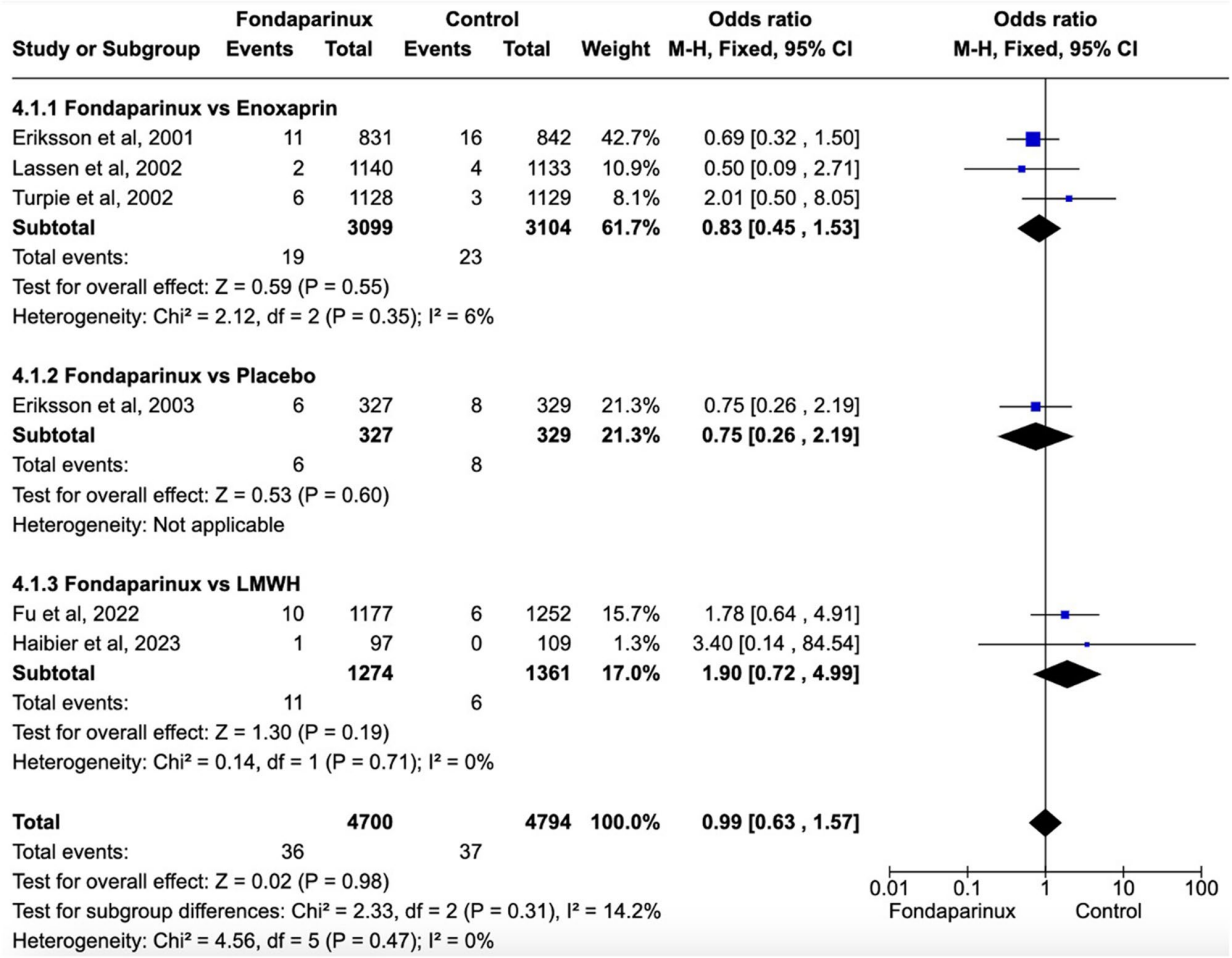
Forest Plot showing effects of Fondaparinux in the incidence of mortality. There were no significant differences in mortality rates between groups

**Fig. 6:**
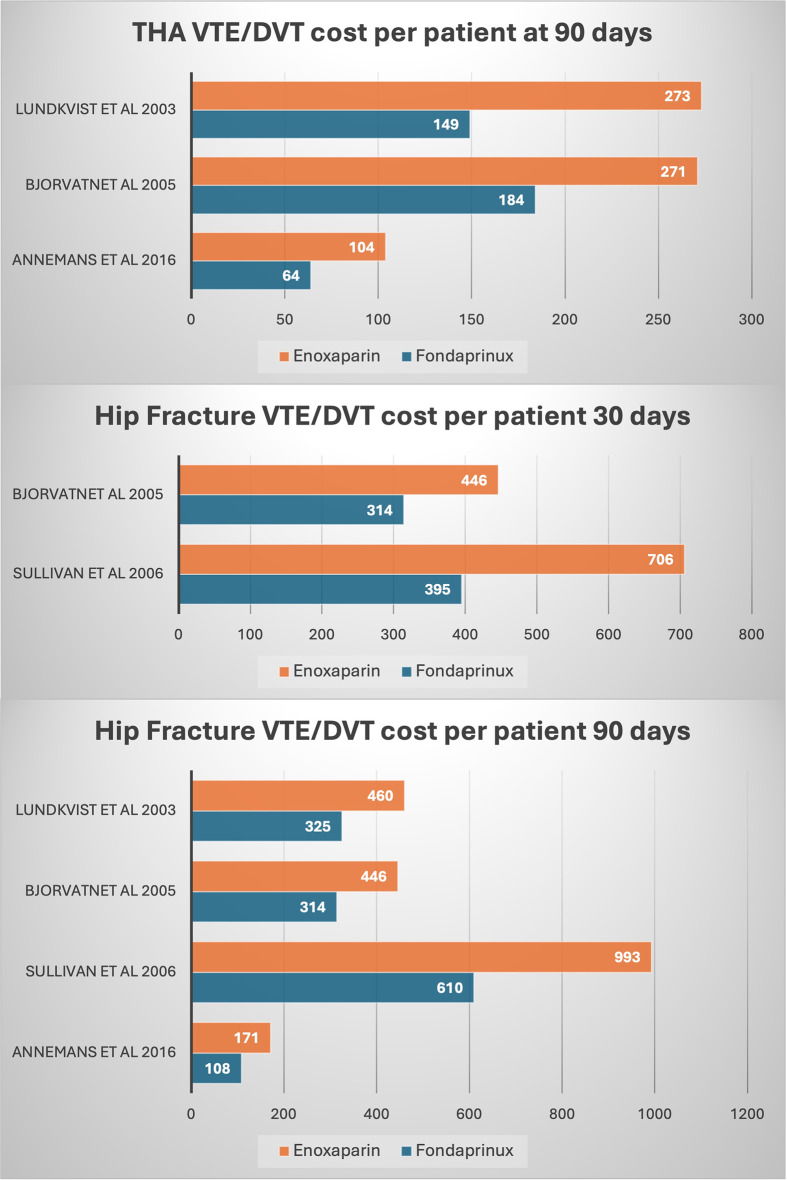
Mean costs per patient per event of VTE/DVT for fondaparinux versus enoxaparin. Depending on the study, the costs were evaluated in euros, pounds, and/or dollars

### Baseline characteristics

Table 1 summarizes the main characteristics of the included studies. There were 15 original studies (*n* = 32534 patients) based on real-world data comparing fondaparinux with other options [9–12, 20–24, 26–30] and four cost studies based on simulation models [25, 31–34], which is why there were no baseline characteristics for them. The studies were primarily conducted in Japan [9, 23–26, 28–30], China [10, 11, 27], and Sweden [18, 19], utilizing both randomized controlled trials (seven studies) [12, 20–23, 25, 26] and cohort designs (eight studies) [9–11, 24, 27–30]. Among these, seven studies compared fondaparinux with enoxaparin [9, 12, 21–24, 30], two with LMWH [10, 11], one with nadroparin [27], six with placebo [9, 20, 23, 25, 26, 30], and two with mechanical prophylaxis [28, 29]. There were three studies with more than two arms that included data for both the enoxaparin and the placebo groups [9, 23, 30]. Four studies focused on patients undergoing surgery after hip fracture, and eleven studies on elective THA [9, 20, 21, 29]. The average age ranged from 58.0 to 83.0 years. The BMI ranged from 21.4 to 28.0, and the dose of fondaparinux used in the studies was 2.5 mg, except for one study that used 0.25 mg. Regarding the doses of enoxaparin, three studies reported 40 mg [12, 21, 23], one study reported 30 mg [22], another 4000IU [30], and one 2000IU [9]. The remaining studies did not report the doses of enoxaparin, nadroparin, or any other type of LMWH. The type of examination and time of evaluation is shown in Additional Table 1.

**Table 1:** Baseline characteristics of included studies

Studies	Region	Study design	Type of hip surgery	Follow-up	Sex M(F)	Age (yrs) Mean (SD) * Median (Range)	BMI (kg/m^2^) Mean (SD) * Median (Range)	History of venous thromboembolism *N* (%)	Dose
**Fondaparinux vs. Enoxaparin**									
Eriksson et al. 2001 [21]	Sweden	RCT	Fracture	6 weeks	187 (644)/224 (618)	76.8 (12.3)/77.3 (12.6)	23.8 (4)/64.2 (13.8)	29 (3.5)/32 (3.8)	FDP: 2.5 mg; EXP: 40 mg
Lassen et al. 2002 [12]	Denmark	RCT	THA	6 weeks	396 (512)/402 (517)	* 67 (30–90)/67 (24–97)	*26 (15–45)/26 (14–51)	35 (4)/ 40 (4)	FDP: 2.5 mg; EXP: 40 mg
Migita et al. 2014 [24]	Japan	Cohort	THA	NR	128 (740)	66.7 (10.5)	23.6 (4.2)	5 (0.6)	FDP: 2.5 mg; EXP: NR
Okada et al. 2015 [30]	Japan	Cohort	THA	7 days	22 (78)/9 (91)	*58 (35–82)/61 (40–86)	*23 (16–40)/24(17–45)	NR	FDP: 2.5 mg; EXP: 4000 UI
Sasaki et al. 2011 [9]	Japan	Cohort	Fracture	14 days	6 (21)/6 (22)	79.7 (11.4)/82.4 (7.4)	21.4 (4.27)/20.68 (3.58)	NR	FDP: 2.5 mg; EXP: 2000 UI
Turpie et al. 2002 [22]	Canada	RCT	THA	6 weeks	556 (572)/522 (607)	*67 (18–92)/*67 (19–91)	*28 (14–73)/*28 (13–83)	52 (5)/ 63 (6)	FDP: 2.5 mg; EXP: 30 mg
Yokote et al. 2011 [23]	Japan	RCT	THA	12 weeks	14 (70)/16 (67)	63 (10)/64 (11)	22.5 (4.8)/23 (3.3)	NR	FDP: 2.5 mg; EXP: 40 mg
**Fondaparinux vs. LMWH**									
Haibier et al. 2023 [10]	China	Cohort	THA	1 month	36 (61)/39 (70)	77.17 (5.48)/76.27 (5.06)	23.76 (4.03)/23.51 (3.54)	NR	FDP: 2.5 mg; LWMH: NR
Wang et al. 2024 [11]	China	Cohort	THA	7 days	10 (20)/14 (16)	71 (2.128)/70.3 (1.714)	NR	NR	FDP: 2.5 mg; LWMH: NR
**Fondaparinux vs. Nadroparin**									
Gao et al. 2024 [27]	China	Cohort	THA	NR	94 (220)/103 (175)	67.06 (10.68)/ 68.85 (10.007)	NR	NR	FDP: 0.25 mg; NDP: Not reported
**Fondaparinux vs. Placebo**									
Eriksson et al. 2003 [20]	Sweden	RCT	Fracture	32 days	92 (235)/98 (231)	*79 (23–94)/*79 (28.96)	*24 (15–38)/*24 (15–41)	13 (4)/12 (3.6)	FDP: 2.5 mg
Fuji et al., 2007 [25]	Japan	RCT	THA	17 days	75 (351)/15 (72)	71 (8)/70.4 (7.9)	NR	NR	FDP: 2.5 mg
Kawaji et al. 2011 [26]	Japan	RCT	THA	14 days	22 (70)/15 (95)	62.4/62.6	24.3/23.6	NR	FDP: 2.5 mg
Okada et al. 2015 [30]	Japan	Cohort	THA	7 days	78 (22)/91 (9)	*58 (35–82)/*61 (40–86)	*23 (16–40)/*24 (17–45)	NR	FDP: 2.5 mg
Sasaki et al. 2011 [9]	Japan	Cohort	Fracture	14 days	6 (21)/5 (24)	79.7 (11.4)/83 (7.5)	21.4 (4.27)/20.83 (3.76)	NR	FDP: 2.5 mg
Yokote et al. 2011 [23]	Japan	RCT	THA	12 weeks	14 (70)/16 (67)	63 (10)/63 (12)	22.5 (4.8)/23.1 (8.1)	NR	FDP: 2.5 mg
**Fondaparinux vs. Mechanical prophylaxis**									
Tsuda et al. 2014 [29]	Japan	Cohort	Fracture	NR	925 (3867)/3639 (14345)	79.2 (9.1)/79.6 (9.4)	NR	NR	NR
Yukizawa et al. 2012 [28]	Japan	Cohort	THA	14 days	27 (76)/20 (47)	58 (8)/68 (8)	24 (5)/24 (6)	6 (0.6)/17 (25.4)	FDP: 2.5 mg

EXP: Enoxaparain; FDP: Fondaparinux; NDP: Nadroparin; NR: Not reported

### Risk of bias

The risk of bias for the six clinical trials is shown in Additional Fig. 1. The studies primarily failed in reporting allocation concealment and in blinding outcome assessments (Additional File 2). Regarding the nine non-randomized studies, their evaluation is detailed in Additional Table 2. These studies were found to be of high quality.

**Table 2:** Bleeding assessment

Effect size	*n* participants	Fixed effect model (OR 95% CI)	I^2^ (%)	*P*-Value
**Minor bleeding**				
Fondaparinux vs. Placebo	1019	OR 4.19, 95% CI 1.17 to 14.92	0	0.03
Fondaparinux vs. Enoxaparin	1928	OR 1.91, 95% CI 1.10 to 3.32	0	0.02
**Bleeding in critical organ**				
Fondaparinux vs. Placebo	819	N/E	N/E	N/E
Fondaparinux vs. Enoxaparin	6203	OR 0.33, 95% CI 0.01 to 8.19	N/A	0.50
**Bleeding leading to reoperation**				
Fondaparinux vs. Placebo	819	OR 1.01, 95% CI 0.14 to 7.19	N/A	1.00
Fondaparinux vs. Enoxaparin	6203	OR 1.43, 95% CI 0.54 to 3.77	0	0.47
**Fatal bleeding**				
Fondaparinux vs. Placebo	875	N/E	N/E	N/E
Fondaparinux vs. Enoxaparin	6258	OR 0.34, 95% CI 0.01 to 8.29	N/A	0.51
**n Transfusions**				
Fondaparinux vs. Placebo	656	OR 1.50, 95% CI 0.83 to 2.72	N/A	0.18
Fondaparinux vs. Enoxaparin	3946	OR 1.11, 95% CI 0.98 to 1.26	24	0.10

N/A: Not applicable; N/E: Not estimable

### Venous thromboembolism

Fondaparinux demonstrated significantly fewer VTE events compared to the control group (OR 0.43, 95% CI 0.31 to 0.61; participants = 29,884; studies = 17; I^2^ = 71%) (Fig. 2). Similarly, a significant difference was observed when compared to LMWHs, including enoxaparin and nadroparin (OR 0.55, 95% CI 0.41 to 0.74; participants = 5,964; studies = 10; I2 = 41%). When compared with placebo or mechanical prophylaxis, fondaparinux significantly reduced the incidence of VTE (OR 0.27, 95% CI 0.11 to 0.62; participants = 23,920; studies = 7; I2 = 84%).

### Deep vein thrombosis

Fondaparinux showed a lower incidence of distal DVT compared to the control group (OR 0.43, 95% CI 0.31 to 0.62; participants = 6,227; studies = 10; I2 = 57%) (Fig. 3a). Fondaparinux also demonstrated a lower incidence of distal DVT compared to enoxaparin (OR 0.49, 95% CI 0.39 to 0.62; participants = 5,306; studies = 6; I2 = 8%) and placebo (OR 0.24, 95% CI 0.07 to 0.77; participants = 921; studies = 4; I2 = 78%).

Regarding proximal DVT, fondaparinux significantly reduced the incidence compared to the control group (OR 0.33, 95% CI 0.15 to 0.75; participants = 6,368; studies = 10; I2 = 66%) (Fig. 3b). Compared with enoxaparin, no significant differences were found (OR 0.48, 95% CI 0.20 to 1.17; participants = 5,422; studies = 6; I2 = 48%). Compared with placebo, fondaparinux significantly decreased the incidence of proximal DVT (OR 0.16, 95% CI 0.04 to 0.61; participants = 9,946; studies = 4; I2 = 48%).

### Pulmonary embolism and mortality

Regarding non-fatal PE, there were no significant differences between groups (OR 1.04, 95% CI 0.55 to 1.97; participants = 31,088; studies = 12; I^2^ = 23%) (Fig. 4a). In terms of fatal PE, there were also no significant differences observed between groups (OR 0.60, 95% CI 0.14 to 2.53; participants = 7,409; studies = 6; I^2^ = 0%) (Fig. 4b). Additionally, there were no significant differences in mortality rates between groups (OR 0.99, 95% CI 0.63 to 1.57; participants = 9,494; studies = 6; I^2^ = 0%) (Fig. 5).

### Bleeding

Minor bleeding was significantly lower in the control group compared with fondaparinux (OR 2.21, 95% CI 1.34 to 3.65; participants = 2,947; studies = 6; I2 = 0%). When divided by subgroups, enoxaparin presented lower minor bleeding (OR 1.91, 95% CI 1.10 to 3.32; participants = 1,928; studies = 3; I2 = 0%) as well as placebo (OR 4.19, 95% CI 1.17 to 14.92; participants = 1,019; studies = 3; I2 = 0%) compared with fondaparinux. There were no significant differences between the groups regarding bleeding in critical organs (OR 0.33, 95% CI 0.01 to 8.19; participants = 7,022; studies = 5; I2 = NA), bleeding leading to reoperation (OR 1.34, 95% CI 0.56 to 3.18; participants = 7,022; studies = 5; I2 = 0%), fatal bleeding (OR 0.34, 95% CI 0.01 to 8.29; participants = 7,133; studies = 7; I2 = NA), or regarding the number of transfusions (OR 1.13, 95% CI 0.99 to 1.28; participants = 4,602; studies = 3; I2 = 12%). Table 2 presents the outcomes related to bleeding.

### Costs

The costs were evaluated in euros, pounds, and/or dollars depending on the study. In elective THA at 90 days, fondaparinux presented a cost of 132 versus 216 for enoxaparin. In hip fracture surgery at 30 days, fondaparinux showed a cost of 355 versus 576 for enoxaparin, while at 90 days post-hip fracture surgery, fondaparinux had a cost of 339 versus 518 for enoxaparin. Figure 6 displays the mean costs per patient per event of VTE/DVT for fondaparinux versus enoxaparin.

### Sensitivity analyses

Upon removing the study with the most weight from all the main variables, all variables maintained the direction of their results except for proximal DVT, which changed from showing no significant differences between groups to showing that fondaparinux significantly reduced the incidence of proximal DVT compared with enoxaparin (OR 0.27, 95% CI 0.16 to 0.47; participants = 3,776; studies = 5; I2 = 0%).

When assessing the effect according to the type of indication (Table 3), fondaparinux showed a reduction in the incidence of VTE and DVT (proximal and distal) in hip fracture surgery compared to LMWHs. On the other hand, in elective total hip arthroplasty (THA), fondaparinux showed a significant reduction in VTE and distal DVT compared to LMWHs, but there were no differences regarding proximal DVT.

**Table 3:** Indication-based sensitivity analysis comparing Fondaparinux vs. LMWHs including Enoxaparin and Nadroparin

Effect size	*n* studies	*n* participants	Fixed effect model (OR 95% CI)	I^2^ (%)	*P*-Value
**Hip Fracture**					
VTE	2	1305	OR 0.37, 95% CI 0.27 to 0.52	0%	< 0.00001
Distal DVT	2	1308	OR 0.39, 95% CI 0.27 to 0.57	0%	< 0.00001
Proximal DVT	2	1351	OR 0.22, 95% CI 0.10 to 0.51	0%	0.0004
**THA**					
VTE	8	4659	OR 0.60, 95% CI 0.48 to 0.75	27%	< 0.0001
Distal DVT	4	3998	OR 0.55, 95% CI 0.42 to 0.73	2%	< 0.0001
Proximal DVT	4	4071	OR 0.65, 95% CI 0.20 to 2.06*	66%	0.46

*:Random effect model

### Publication bias

Publication bias is demonstrated in Additional File 3. There was asymmetry and clustering of studies at the upper end of the funnel plot, indicating the presence of publication bias.

### Grade

According to the GRADE framework, VTE, proximal DVT, and fatal bleeding were found to have moderate certainty. Distal DVT demonstrated high certainty, while non-fatal PE showed very low certainty. The studies exhibited publication bias, and for some variables, most of the study designs were non-randomized. Table 4 displays the certainty analysis.

**Table 4:** GRADE assessment

Certainty assessment							№ of patients		Effect		Certainty	Importance
№ of studies	Study design	Risk of bias	Inconsistency	Indirectness	Imprecision	Other considerations	Fondaparinux	Control	Relative (95% CI)	Absolute (95% CI)		
**VTE**												
17	non-randomised studies	not serious	not serious	not serious	not serious	publication bias strongly suspected^a^	258/8413 (3.1%)	653/21,471 (3.0%)	**OR 0.43** (0.31 to 0.61)	**17 fewer per 1000** (from 21 fewer to 12 fewer)	⨁⨁⨁◯ Moderate^a^	CRITICAL
**Distal DVT**												
10	randomised trials	not serious	not serious	not serious	not serious	publication bias strongly suspected^a^	176/3192 (5.5%)	357/3035 (11.8%)	**OR 0.43** (0.30 to 0.61)	**63 fewer per 1000** (from 79 fewer to 42 fewer)	⨁⨁⨁⨁ High^a^	CRITICAL
**Proximal DVT**												
10	randomised trials	not serious	not serious	not serious	not serious	publication bias strongly suspected^a^	36/3262 (1.1%)	115/3106 (3.7%)	**OR 0.33** (0.15 to 0.75)	**24 fewer per 1000** (from 31 fewer to 9 fewer)	⨁⨁⨁◯ Moderate^a^	CRITICAL
**Non-fatal PE**												
12	non-randomised studies	not serious	serious^b^	not serious	not serious	publication bias strongly suspected^a^	48/8993 (0.5%)	172/22,095 (0.8%)	**OR 1.04** (0.55 to 1.97)	**0 fewer per 1000** (from 3 fewer to 7 more)	⨁◯◯◯ Very low^a, b^	CRITICAL
**Fatal Bleeding**												
7	randomised trials	not serious	not serious	not serious	serious^c^	none	0/3561 (0.0%)	1/3572 (0.0%)	**OR 0.34** (0.01 to 8.29)	**0 fewer per 1000** (from 0 fewer to 2 more)	⨁⨁⨁◯ Moderate^c^	CRITICAL

(a) Suspicion of publication bias when visualizing funnel plots; (b) Results show wide variability or unexplained heterogeneity; (c) Wide confidence intervals; CI. confidence interval; OR. odds ratio

## Discussion

According to the present meta-analysis, fondaparinux reduced VTE in individuals undergoing THA and those with hip fractures more effectively than LMWH. Remarkably, fondaparinux continuously demonstrated higher effectiveness in lowering proximal and distal DVT. This effect, which was particularly noticeable in cases of hip fractures, was seen in patients having hip fracture surgery as well as those getting elective THA. The risk of clinically severe bleeding events did not differ significantly. In addition, fondaparinux was less expensive per patient than enoxaparin for thromboembolic events.

The physiological justification for the efficacy of Fondaparinux, a selective inhibitor of Factor Xa, a crucial participant in the coagulation cascade, is provided by its mode of action [35]. The synthetic pentasaccharide fondaparinux binds specifically to antithrombin III, boosting its activity to inhibit just Factor Xa rather than thrombin (Factor IIa), which is different from how heparins work [36]. This selective action allows for more precise and effective coagulation inhibition by reducing thrombus formation without significantly altering bleeding time.

Notable characteristics of fondaparinux’s synthetic chemical composition include a quick onset of action, a lengthy half-life, direct renal excretion and 100% bioavailability when administered subcutaneously [35].

In our study, when only studies that conducted venography were assessed, fondaparinux showed a significantly lower risk of VTE, with a risk ratio of 0.30 (0.17–0.54) compared to enoxaparin. In contrast, when compared with studies that used ultrasound to evaluate VTE, fondaparinux showed a reduced risk of VTE but to a lesser extent compared to enoxaparin, with a risk ratio of 0.55 (0.30–1.01). This may indicate that when more precise diagnostic tests are used, fondaparinux demonstrates a greater reduction in VTE risk. This suggests that the diagnostic methodology could influence the results. This is logical since venography is the standard in diagnosing DVT [37, 38].

On the other hand, when evaluating the influence of the timing of evaluation, it did not appear to affect the results. Although most diagnostic tests were conducted between the 5th and 11th day.

In our study, potential confounding factors were analyzed to assess whether they were balanced between the experimental and control groups. For example, it has been observed that patients with a higher BMI are at greater risk of VTE [39]. In our study, no differences in BMI were observed between the control and experimental groups. Similarly, other parameters that have been shown to increase the risk of thromboembolism, such as increased intraoperative bleeding, surgery duration, or renal function [40–42], showed no differences between the two groups, indicating that both started from a similar baseline. Additionally, other factors such as gender and age, which have also been shown to influence thromboembolism, with it being more common in men and older individuals, were evenly distributed across both groups. There were also no differences in the duration of treatment between groups.

Despite its potent anticoagulant activity, fondaparinux maintains a safety profile comparable to that of low molecular weight heparins, due to its selectivity which reduces the risk of hemorrhagic complications. Previous studies have identified several factors that increase the risk of bleeding with fondaparinux, including male gender and low body weight [43]. Specifically, a body weight under 50 kg and moderate renal insufficiency can increase drug exposure, thereby elevating the risk of bleeding [44]. On the contrary, the involvement of an orthogeriatrician has been shown to improve discharge optimization in patients with hip fractures, resulting in better hemoglobin levels at discharge [45].

In these studies, patient comfort or satisfaction with the different options was not analyzed due to the lack of studies reporting these outcomes. Generally, fondaparinux is seen as easier to manage by the patient because it is administered at a fixed dose, does not require monitoring, and reduces the incidence of subcutaneous hemorrhage [36].

Wang et al. observed that subcutaneous ecchymosis was 3.3% on the seventh day in the fondaparinux group compared to 20.0% in the LMWH group, reaching significant differences [11]. Additionally, the circumference of swelling was also significantly smaller. Wang et al. also observed less pain on the seventh day, assessed using the VAS scale in the fondaparinux group, with no differences found on the third and fifth days.

Another important aspect to consider is treatment compliance. In a recent study [46], 73% of patients treated with fondaparinux showed a preference for the subcutaneous route of administration. On the other hand, approximately 85% of patients who received new oral anticoagulants—which have also demonstrated favorable outcomes in total hip arthroplasty—preferred this form of administration [47]. Furthermore, a high level of treatment compliance was observed, reaching 99% across all groups, regardless of the route of administration used.

The lower cost of fondaparinux as compared to enoxaparin, may be explained by the effectiveness of reducing thromboembolic events, which can minimize the costs of treating these consequences [48]. In particular, the fondaparinux group spent an average of 132 euros per patient per VTE/DVT event in THA at 90 days, while enoxaparin cost 216 euros. The average cost per patient for a VTE/DVT event in the case of a hip fracture at 30 days was $355, whereas the enoxaparin group’s cost was $576. Additionally, the cost per patient for a VTE/DVT incident at 90 days after a hip fracture was 339 (euros/dollars), while the cost for enoxaparin was 518. The extended hospital stays, and increased resource usage may help to explain this.

It is essential to consider any potential interactions or confounding factors pertaining to the antithrombotic under analysis that might have impacted the outcomes in addition to the previously mentioned factors. The effectiveness and safety of thromboprophylaxis can be impacted by a variety of drugs, nutraceuticals, and diets, including fondaparinux and LMWHs [49].

Compared to LMWHs and other anticoagulants, fondaparinux, exhibits fewer medication interactions. However, the risk of bleeding is also increased by concurrent use of other medications that influence hemostasis, such as nonsteroidal anti-inflammatory drugs (NSAIDs), especially when these medications are taken with platelet inhibitors (e.g., clopidogrel, aspirin) [50].

The risk of bleeding during LMWH medication may be substantially increased by concurrent use of NSAIDs, SSRIs, and SNRIs [51].

Several differences were found between our study’s results and those of other meta-analyses on the subject. For example, Hur et al. discovered fondaparinux was linked to a greater risk of bleeding than LMWH after examining just four studies [13]. On the other hand, our meta-analysis found no significant differences between the groups and differentiated between small and extensive bleeding. Li et al. also noted that fondaparinux enhanced the risk of hemorrhage [52]. Turpie et al. presented the idea of clinically meaningful bleeding in another meta-analysis [53], however they found no distinction between fondaparinux and enoxaparin. Huang et al. reached to the conclusion that fondaparinux was superior to LMWH in lowering VTE; however, their research was constrained because it only examined THA without examining hip fractures and neglected to take into consideration all of the variables [14], we thought of in order to control for heterogeneity. Only two fondaparinux trials were included in the most current meta-analysis, which was carried out by Migliorini et al. in 2024 [47]. Our results, which also included cost analysis after prophylaxis in hip fracture surgery, are consistent with a meta-analysis of cost analysis studies conducted by Dranitsaris et al., which found fondaparinux to be more cost-effective than enoxaparin in antithrombotic prophylaxis following THA and TKA [54]. These fondaparinux results align with other indications, such total knee replacement [55].

Most studies utilized the approved dosage of 2.5 mg as stable and recommended by the European Medicines Agency (EMA) [56]. For patients with moderate renal impairment, the dose should be reduced to 1.5 mg once daily [57].

### Limitations

This study has several limitations. The research encompassed a variety of study designs, both randomized and non-randomized. Additionally, the small number of studies within certain subgroups curtailed the robustness of sensitivity analyses and the ability to achieve consistent results across different settings and populations. The inability to perform meta-regression to adjust for confounding variables or external influences further limited the interpretability of the data. The original studies did not assess whether a tourniquet was used during THA [58]. The assessment of publication bias relied solely on visual inspection of funnel plots due to software constraints (Review Manager 5.4.1), which does not support statistical tests like Egger’s regression. A significant limitation across the studies was the uniform use of a 2.5 mg dose, which is the standard recommended dosage but restricted the assessment of the efficacy and safety of alternative dosing regimens. This uniformity in dosing precludes any analysis on dose-response relationships that could be crucial for optimizing treatment protocols. Information gaps regarding clinically significant bleeding outcomes were also evident; many studies did not sufficiently report how bleeding events impacted patient health or the timing of these events, which are critical for understanding the risk profile of the treatment. Other clinically relevant variables such as pain from injections or specific analytical values were rarely assessed or reported inconsistently, making it difficult to compare these factors statistically across studies. Cost assessments were particularly challenging due to the lack of dispersion data in the studies, forcing reliance on estimation models or Cochrane rules for approximations. Some studies did not specify the total number of participants at the point when variables were measured, necessitating the use of initial participant totals for calculations. Additionally, there was a lack of specified follow-up periods, which is crucial when assessing the timing and impact of certain events. Most studies have focused on fractures, including various types of treatments, ranging from intramedullary nail fixation to prostheses, and the results were not divided. This lack of division in the results could introduce heterogeneity into the interpretation of the findings. Finally, there was a lack of comparative evidence between new oral anticoagulants and fondaparinux.

## Conclusion

Based on the results from this meta-analysis, fondaparinux significantly reduces the incidence of venous VTE and distal DVT, compared to controls and LMWHs in patients undergoing elective THA and hip fracture surgery. Fondaparinux maintains a safety profile comparable to enoxaparin, with no significant differences in clinically significant bleeding. Additionally, fondaparinux is more cost-effective, presenting lower average costs per thromboembolic event per patient than enoxaparin. These findings support the use of fondaparinux as a well-tolerated, effective, and economically advantageous alternative for thromboprophylaxis in hip surgery.

## Electronic supplementary material

Below is the link to the electronic supplementary material.

Supplementary Material 1

Supplementary Material 2

Supplementary Material 3

Supplementary Material 4

Supplementary Material 5

Supplementary Material 6

## Data Availability

No datasets were generated or analysed during the current study.

## References

[1] Villalón-Rubio D, García-Tercero E, López-Gómez J, González H, Belenguer-Varea Á, Cunha-Pérez C, et al. Exploring factors influencing delirium incidence: insights from the Alzira cohort study, 2012–2021. Rev Esp Geriatr Gerontol. 2024;60(1):101571. 10.1016/j.regg.2024.101571. 10.1016/j.regg.2024.101571 39642399

[2] Pabinger C, Geissler A. Utilization rates of hip arthroplasty in OECD countries. Osteoarthritis Cartilage. 2014;22(6):734–41. 10.1016/j.joca.2014.04.009. 24780823 10.1016/j.joca.2014.04.009

[3] Maffulli N, Aicale R. Proximal femoral fractures in the elderly: A few things to know, and some to forget. Med (Kaunas). 2022;58(10):1314. 10.3390/medicina58101314. 10.3390/medicina58101314 PMC9612001 36295475

[4] Harris E, Clement N, MacLullich A, Farrow L. The impact of an ageing population on future increases in hip fracture burden. Bone Joint J. 2024;106–B(1):62–8. 10.1302/0301-620X.106B1.BJJ-2023-0740. 10.1302/0301-620X.106B1.BJJ-2023-0740.R1 38160690

[5] Kwong LM. Hip fracture and venous thromboembolism in the elderly. J Surg Orthop Adv. 2004;13(3):139–48. 15559689

[6] Beauchamp-Chalifour P, Belzile ÉL, Michael R, Langevin V, Gaudreau N, Normandeau N, et al. The risk of venous thromboembolism in surgically treated hip fracture: A retrospective cohort study of 5184 patients. Orthop Traumatol Surg Res. 2022;108(1):103142. 10.1016/j.otsr.2021.103142. 34775033 10.1016/j.otsr.2021.103142

[7] Rosencher N, Vielpeau C, Emmerich J, Fagnani F, Samama CM, ESCORTE group. Venous thromboembolism and mortality after hip fracture surgery: the ESCORTE study. J Thromb Haemost. 2005;3(9):2006–14. 10.1111/j.1538-7836.2005.01545.x. 16102107 10.1111/j.1538-7836.2005.01545.x

[8] Martin KA, Molsberry R, Cuttica MJ, Desai KR, Schimmel DR, Khan SS. Time trends in pulmonary embolism mortality rates in the united States, 1999 to 2018. J Am Heart Assoc. 2020;9(17):e016784. 10.1161/JAHA.120.016784. 32809909 10.1161/JAHA.120.016784 PMC7660782

[9] Sasaki S, Miyakoshi N, Matsuura H, Saito H, Nakanishi T, Kudo Y, et al. Prospective study on the efficacies of Fondaparinux and Enoxaparin in preventing venous thromboembolism after hip fracture surgery. J Orthop Sci. 2011;16(1):64–70. 10.1007/s00776-010-0011-5. 21293896 10.1007/s00776-010-0011-5

[10] Haibier A, Yusufu A, Lin H, Kayierhan A, Abudukelimu Y, Abudurexiti T. Efficacy and safety study of Low-Molecular-Weight heparin and Fondaparinux sodium after hip arthroplasty: A retrospective cohort study. Orthop Res Rev. 2023;15:253–61. 10.2147/ORR.S431372. 38033454 10.2147/ORR.S431372 PMC10684995

[11] Wang L, Xu Z, Zhang L. A comparison between Fondaparinux sodium and Low-Molecular-Weight heparin in preventing patients undergoing hip replacement from deep vein thrombosis. J Musculoskelet Neuronal Interact. 2024;24(2):185–91. 38826001 PMC11145315

[12] Lassen MR, Bauer KA, Eriksson BI, Turpie AG, European Pentasaccharide Elective Surgery Study (EPHESUS) Steering Committee. Postoperative Fondaparinux versus preoperative Enoxaparin for prevention of venous thromboembolism in elective hip-replacement surgery: a randomised double-blind comparison. Lancet. 2002;359(9319):1715–20. 10.1016/S0140-6736(02)08652-X. 12049858 10.1016/S0140-6736(02)08652-X

[13] Hur M, Park SK, Koo CH, Jung ED, Kang P, Kim WH, et al. Comparative efficacy and safety of anticoagulants for prevention of venous thromboembolism after hip and knee arthroplasty. Acta Orthop. 2017;88(6):634–41. 10.1080/17453674.2017.1361131. 28787226 10.1080/17453674.2017.1361131 PMC5694808

[14] Huang Z, Xu X, Xu D, Zhao P, Zou M. Efficacy of 11 anticoagulants for the prevention of venous thromboembolism after total hip or knee arthroplasty: A systematic review and network meta-analysis. Med (Baltim). 2023;102(2):e32635. 10.1097/MD.0000000000032635. 10.1097/MD.0000000000032635 PMC9839234 36637921

[15] Page MJ, McKenzie JE, Bossuyt PM, Boutron I, Hoffmann TC, Mulrow CD, et al. The PRISMA 2020 statement: an updated guideline for reporting systematic reviews. BMJ. 2021;372:n71. 10.1136/bmj.n71. 33782057 10.1136/bmj.n71 PMC8005924

[16] Slim K, Nini E, Forestier D, Kwiatkowski F, Panis Y, Chipponi J. Methodological index for non-randomized studies (minors): development and validation of a new instrument. ANZ J Surg. 2003;73:712–6. 10.1046/j.1445-2197.2003.02748.x. 12956787 10.1046/j.1445-2197.2003.02748.x

[17] Sterne JAC, Savović J, Page MJ, Elbers RG, Blencowe NS, Boutron I, et al. RoB 2: a revised tool for assessing risk of bias in randomised trials. BMJ. 2019;366:l4898. 31462531 10.1136/bmj.l4898

[18] Higgins JP, Thomas J, Chandler J, Cumpston M, Li T, Page MJ, et al. Cochrane handbook for systematic reviews of interventions. Wiley; 2019. 10.1002/14651858.ED000142 PMC10284251 31643080

[19] Guyatt GH, Thorlund K, Oxman AD, Walter SD, Patrick D, Furukawa TA, Johnston BC, et al. GRADE guidelines: 13. Preparing summary of findings tables and evidence profiles-continuous outcomes. J Clin Epidemiol. 2013;66:173–83. 23116689 10.1016/j.jclinepi.2012.08.001

[20] Eriksson BI, Lassen MR. PENTasaccharide in HIp-FRActure surgery plus investigators. Duration of prophylaxis against venous thromboembolism with Fondaparinux after hip fracture surgery: a multicenter, randomized, placebo-controlled, double-blind study. Arch Intern Med. 2003;163(11):1337–42. 10.1001/archinte.163.11.1337. 12796070 10.1001/archinte.163.11.1337

[21] Eriksson BI, Bauer KA, Lassen MR, Turpie AG. Steering committee of the pentasaccharide in Hip-Fracture surgery study. Fondaparinux compared with Enoxaparin for the prevention of venous thromboembolism after hip-fracture surgery. N Engl J Med. 2001;345(18):1298–304. 10.1056/NEJMoa011100. 11794148 10.1056/NEJMoa011100

[22] Turpie AG, Bauer KA, Eriksson BI, Lassen MR, PENTATHALON 2000 Study Steering Committee. Postoperative fondaparinux versus postoperative enoxaparin for prevention of venous thromboembolism after elective hip-replacement surgery: a randomised double-blind trial. Lancet. 2002;359(9319):1721-6. 10.1016/S0140-6736(02)08648-8. Erratum in: Lancet 2002;360(9339):1102. 10.1016/S0140-6736(02)08648-8 12049860

[23] Yokote R, Matsubara M, Hirasawa N, Hagio S, Ishii K, Takata C. Is routine chemical thromboprophylaxis after total hip replacement really necessary in a Japanese population? J Bone Joint Surg Br. 2011;93(2):251–6. 10.1302/0301-620X.93B2.25795. 21282767 10.1302/0301-620X.93B2.25795

[24] Migita K, Bito S, Nakamura M, Miyata S, Saito M, Kakizaki H, et al. Venous thromboembolism after total joint arthroplasty: results from a Japanese multicenter cohort study. Arthritis Res Ther. 2014;16(4):R154. 10.1186/ar4616. 25047862 10.1186/ar4616 PMC4223565

[25] Fuji T, Fujita S, Ochi T. Fondaparinux prevents venous thromboembolism after joint replacement surgery in Japanese patients. Int Orthop. 2008;32(4):443– 51. 10.1007/s00264-007-0360-7. Epub 2007 Apr 28. PMID: 17468868. 10.1007/s00264-007-0360-7 PMC2532275 17468868

[26] Kawaji H, Ishii M, Tamaki Y, Hamasaki M, Ishikawa H, Sasaki K, et al. Postoperative prophylactic effect of Fondaparinux for prevention of deep venous thrombosis after cemented total hip replacement: a comparative study. Mod Rheumatol. 2012;22(2):216–22. 10.1007/s10165-011-0496-6. 21761228 10.1007/s10165-011-0496-6

[27] Gao X, Jin X, Huang R, Li Z, Zhang H, Fan P. Comparison of efficacy of Nadroparin and Fondaparinux sodium for prevention of deep vein thromboembolism in lower extremities after total hip arthroplasty and total knee arthroplasty: a retrospective study of 592 patients. BMC Surg. 2024;24(1):162. 10.1186/s12893-024-02440-0. 38762739 10.1186/s12893-024-02440-0 PMC11102291

[28] Yukizawa Y, Inaba Y, Watanabe S, Yajima S, Kobayashi N, Ishida T, Iwamoto N, et al. Association between venous thromboembolism and plasma levels of both soluble fibrin and plasminogen-activator inhibitor 1 in 170 patients undergoing total hip arthroplasty. Acta Orthop. 2012;83(1):14–21. 10.3109/17453674.2011.652886. 22248164 10.3109/17453674.2011.652886 PMC3278651

[29] Tsuda Y, Yasunaga H, Horiguchi H, Fushimi K, Kawano H, Tanaka S. Effects of Fondaparinux on pulmonary embolism following hemiarthroplasty for femoral neck fracture: a retrospective observational study using the Japanese diagnosis procedure combination database. J Orthop Sci. 2014;19(6):991–6. 10.1007/s00776-014-0607-2. 25034972 10.1007/s00776-014-0607-2

[30] Okada Y, Endo H, Mitani S, Fujiwara K, Tetsunaga T, Kagawa Y et al. Venous thromboembolism after total hip arthroplasty diagnosed by enhanced computed tomography:comparison of selective thromboprophylaxis and no thromboprophylaxis. Acta Med Okayama. 2015;69(4):205– 12. doi: 10.18926/AMO/53556. Erratum in: Acta Med Okayama. 2015;69(5):325. 10.18926/AMO/53680 10.18926/AMO/53556 26289911

[31] Annemans L, Minjoulat-Rey MC, De Knock M, Vranckx K, Czarka M, Gabriel S, et al. Cost consequence analysis of Fondaparinux versus Enoxaparin in the prevention of venous thromboembolism after major orthopaedic surgery in Belgium. Acta Clin Belg. 2004;59(6):346–57. 10.1179/acb.2004.050. 15819379 10.1179/acb.2004.050

[32] Sullivan SD, Kwong L, Nutescu E. Cost-effectiveness of Fondaparinux compared with Enoxaparin as prophylaxis against venous thromboembolism in patients undergoing hip fracture surgery. Value Health 2006 Mar-Apr;9(2):68–76. 10.1111/j.1524-4733.2006.00085.x 10.1111/j.1524-4733.2006.00085.x 16626410

[33] Bjorvatn A, Kristiansen F. Fondaparinux sodium compared with Enoxaparin sodium: a cost-effectiveness analysis. Am J Cardiovasc Drugs. 2005;5(2):121–30. 10.2165/00129784-200505020-00006. 15725043 10.2165/00129784-200505020-00006

[34] Lundkvist J, Bergqvist D, Jönsson B. Cost-effectiveness of Fondaparinux vs. enoxaparin as venous thromboembolism prophylaxis in Sweden. Eur J Health Econ. 2003;4(4):254–62. 10.1007/s10198-003-0175-4. 15609193 10.1007/s10198-003-0175-4

[35] Bauersachs RM. Fondaparinux sodium: recent advances in the management of thrombosis. J Cardiovasc Pharmacol Ther. 2023;28:10742484221145010. 10.1177/10742484221145010. 36594404 10.1177/10742484221145010

[36] Shorr AF. The pharmacoeconomics of deep vein thrombosis treatment. Am J Med. 2007;120(10 Suppl 2):S35–41. 10.1016/j.amjmed.2007.08.012. 17916458 10.1016/j.amjmed.2007.08.012

[37] Theerakulpisut D, Wongsurawat N, Somboonporn C. Detection of lower limb deep vein thrombosis: comparison between radionuclide venography and venous ultrasonography. World J Nucl Med. 2018;17(1):27–33. 10.4103/wjnm.WJNM_13_17. 29398962 10.4103/wjnm.WJNM_13_17 PMC5778710

[38] Douketis JD, Ginsberg JS. Diagnosis of deep vein thrombosis. Can Fam Physician. 1996;42:497–503. 8616289 PMC2146310

[39] Gurunathan U, Barras M, McDougall C, Nandurkar H, Eley V. Obesity and the risk of venous thromboembolism after major lower limb orthopaedic surgery: A literature review. Thromb Haemost. 2022;122(12):1969–79. 10.1055/s-0042-1757200. 36384225 10.1055/s-0042-1757200

[40] Liu KC, Piple AS, Richardson MK, Mayer LW, Mayfield CK, Christ AB, et al. Increased risk of venous thromboembolism in patients with postoperative Anemia after total joint arthroplasty: are transfusions to blame?? J Bone Joint Surg Am. 2023;105(17):1354–61. 10.2106/JBJS.23.00146. 37471565 10.2106/JBJS.23.00146

[41] Gu A, Wei C, Chen AZ, Malahias MA, Fassihi SC, Ast MP, et al. Operative time greater than 120 minutes is associated with increased pulmonary and thromboembolic complications following revision total hip arthroplasty. Eur J Orthop Surg Traumatol. 2020;30(8):1393–400. 10.1007/s00590-020-02712-4. 32524203 10.1007/s00590-020-02712-4

[42] Zeng Y, Shen B, Yang J, Zhou Z, Kang P, Pei F. Preoperative comorbidities as potential risk factors for venous thromboembolism after joint arthroplasty: a systematic review and meta-analysis of cohort and case-control studies. J Arthroplasty. 2014;29(12):2430–8. 10.1016/j.arth.2014.05.018. 24996584 10.1016/j.arth.2014.05.018

[43] Zufferey PJ, Ollier E, Delavenne X, Laporte S, Mismetti P, Duffull SB. Incidence and risk factors of major bleeding following major orthopaedic surgery with Fondaparinux thromboprophylaxis. A time-to-event analysis. Br J Clin Pharmacol. 2018;84(10):2242–51. 10.1111/bcp.13663. 29877590 10.1111/bcp.13663 PMC6138479

[44] Delavenne X, Zufferey P, Baylot D, Nguyen P, Borg JY, Fontenay M, et al. Population pharmacokinetics of Fondaparinux administered at prophylactic doses after major orthopaedic surgery in everyday practice. Thromb Haemost. 2010;104(2):252–60. 10.1160/TH10-02-0127. 20539905 10.1160/TH10-02-0127

[45] Quaranta M, Miranda L, Oliva F, Migliorini F, Pezzuti G, Maffulli N. Haemoglobin and transfusions in elderly patients with hip fractures: the effect of a dedicated orthogeriatrician. J Orthop Surg Res. 2021;16(1):387. 10.1186/s13018-021-02524-0. 34134743 10.1186/s13018-021-02524-0 PMC8207795

[46] DI Benedetto P, Vetrugno L, DE Franceschi D, Gisonni R, Causero A, Rocca GD. Patient compliance with new oral anticoagulants after major orthopaedic surgery: Rivaroxaban and Dabigatran compared with subcutaneous injection of Fondaparinux. Joints. 2017;4(4):214–21. 10.11138/jts/2016.4.4.214. 28217657 10.11138/jts/2016.4.4.214 PMC5297345

[47] Migliorini F, Maffulli N, Velaj E, Bell A, Kämmer D, Hildebrand F, et al. Antithrombotic prophylaxis following total hip arthroplasty: a level I bayesian network meta-analysis. J Orthop Traumatol. 2024;25(1):1. 10.1186/s10195-023-00742-2. 38194191 10.1186/s10195-023-00742-2 PMC10776533

[48] Ollendorf DA, Vera-Llonch M, Oster G. Cost of venous thromboembolism following major orthopedic surgery in hospitalized patients. Am J Health Syst Pharm. 2002;59(18):1750–4. 10.1093/ajhp/59.18.1750. 12298113 10.1093/ajhp/59.18.1750

[49] Ollier C, Faaij RA, Santoni A, Duvauchelle T, van Haard PM, Schoemaker RC, et al. Absence of interaction of Fondaparinux sodium with aspirin and piroxicam in healthy male volunteers. Clin Pharmacokinet. 2002;41(Suppl 2):31–7. 10.2165/00003088-200241002-00005. 10.2165/00003088-200241002-00005 12383043

[50] Brockow K, Wurpts G, Trautmann A, Pfützner W, Treudler R, Bircher AJ, et al. Guideline for allergological diagnosis of drug hypersensitivity reactions: S2k guideline of the German society for allergology and clinical immunology (DGAKI) in Cooperation with the German dermatological society (DDG), the association of German allergologists (ÄDA), the German society for pediatric allergology (GPA), the German contact dermatitis research group (DKG), the German society for pneumology (DGP), the German society of otorhinolaryngology, head and neck surgery, the Austrian society of allergology and immunology (ÖGAI), the Austrian society of dermatology and venereology (ÖGDV), the German academy of allergology and environmental medicine (DAAU), and the German Documentation center for severe skin reactions (dZh). Allergol Select. 2023;7:122–39. 10.5414/ALX02422E. 37705676 10.5414/ALX02422E PMC10495942

[51] Hirsh J, Bauer KA, Donati MB, Gould M, Samama MM, Weitz JI. Parenteral anticoagulants: American College of Chest Physicians Evidence-Based Clinical Practice Guidelines (8th Edition). Chest. 2008;133(6 Suppl):141S-159S. 10.1378/chest.08-0689. Erratum in: Chest. 2008;134(2):473. 10.1378/chest.08-0689 18574264

[52] Li H, Wang J, Xiao J, Shi Z. [Efficacy and safety of Fondaparinux versus Enoxaparin for preventing venous thromboembolism after major orthopedic surgery: a meta-analysis]. Nan Fang Yi Ke Da Xue Xue Bao. 2013;33(3):370–5. Chinese. 23529234

[53] Turpie AG, Bauer KA, Eriksson BI, Lassen MR. Fondaparinux vs Enoxaparin for the prevention of venous thromboembolism in major orthopedic surgery: a meta-analysis of 4 randomized double-blind studies. Arch Intern Med. 2002;162(16):1833–40. 10.1001/archinte.162.16.1833. 12196081 10.1001/archinte.162.16.1833

[54] Dranitsaris G, Kahn SR, Stumpo C, Paton TW, Martineau J, Smith R, et al. Pharmacoeconomic analysis of Fondaparinux versus Enoxaparin for the prevention of thromboembolic events in orthopedic surgery patients. Am J Cardiovasc Drugs. 2004;4(5):325–33. 10.2165/00129784-200404050-00005. 15449974 10.2165/00129784-200404050-00005

[55] Yang T, Liu Z, Zhang B, Zhang J, Ma A, Cao D, et al. Comparison of the efficacy of low-molecular-weight heparin and Fondaparinux sodium after total knee arthroplasty: a retrospective cohort study. BMC Musculoskelet Disord. 2023;24(1):552. 10.1186/s12891-023-06674-6. 37403062 10.1186/s12891-023-06674-6 PMC10320998

[56] Arixtra. Fondaparinux sodium. Summary of Product Characteristics. European Medicines Agency. Available at: https://www.ema.europa.eu/en/medicines/human/EPAR/arixtra#product-details

[57] Turpie AGG, Lensing AW, Fuji T, Boyle DA. Pharmacokinetic and clinical data supporting the use of Fondaparinux 1.5 mg once daily in the prevention of venous thromboembolism in renally impaired patients. Blood Coagul Fibrinolysis. 2009;20:114–21. 19339838 10.1097/MBC.0b013e328323da86

[58] Migliorini F, Maffulli N, Eschweiler J, Knobe M, Tingart M, Betsch M. Tourniquet use during knee arthroplasty: A bayesian network meta-analysis on pain, function, and thromboembolism. Surgeon. 2022;20(4):241–51. 10.1016/j.surge.2021.03.004. 33967006 10.1016/j.surge.2021.03.004

