# Supplementary figures and images for: Efficacy and safety of fondaparinux in elective total hip arthroplasty and hip fracture surgery: a systematic review and meta-analysis
Source: J Orthop Surg Res. 2025 May 29;20:538. doi: 10.1186/s13018-025-05950-6 (PMC12121286; doi:10.1186/s13018-025-05950-6)

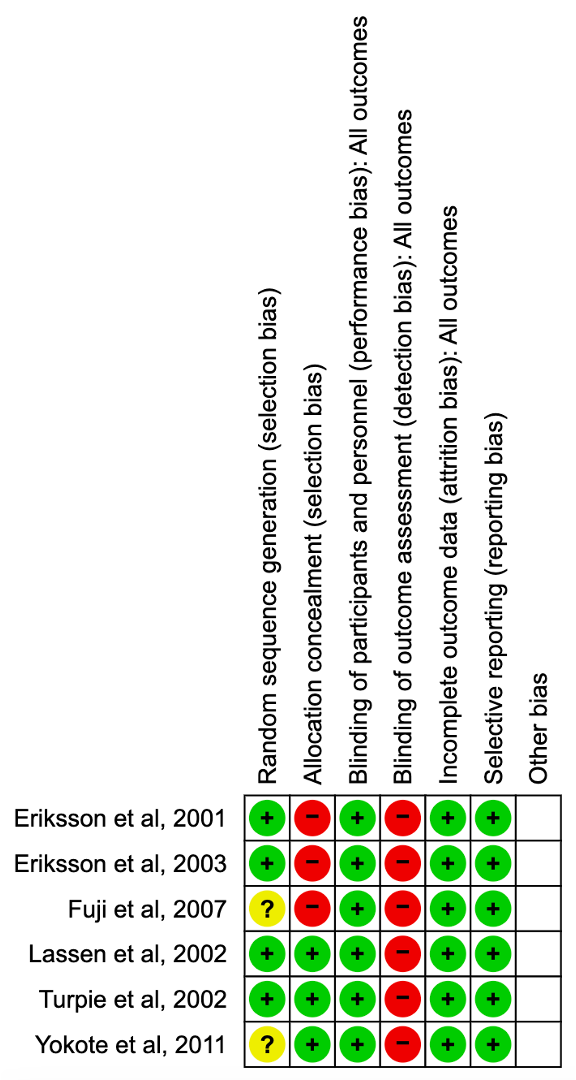

Supplement: Supplementary file 1 — Supplementary Material 1 [file 13018_2025_5950_MOESM1_ESM.png]
