# Supplementary material for: Efficacy and safety of fondaparinux in elective total hip arthroplasty and hip fracture surgery: a systematic review and meta-analysis
Source: J Orthop Surg Res. 2025 May 29;20:538. doi: 10.1186/s13018-025-05950-6 (PMC12121286; doi:10.1186/s13018-025-05950-6)
Supplement: Supplementary file 2 — Supplementary Material 2 [file 13018_2025_5950_MOESM2_ESM.docx]

**Additional File 2.** Justification of the risk of bias in RCTs.

**Eriksson et al, 2001**

A) A computer-generated randomization list.

B) Not reported.

C) Double-blinded.

D) Double-blinded.

E) Reported on tables.

F) All kind of outcomes.

**Eriksson et al, 2003**

A) A computer-generated randomization list.

B) Not reported.

C) Double-blinded.

D) Double-blinded.

E) Reported on tables.

F) All kind of outcomes.

**Fuji et al, 2007**

A) Not well reported.

B) Not reported.

C) Double-blinded.

D) Double-blinded.

E) Reported on tables.

F) All kind of outcomes.

**Lassen et al, 2002**

A) A computer-generated randomization.

B) Figure 1.

C) Double-blinded.

D) Double-blinded.

E) After the treatment period, 28 (2%) of 1140 patients in the fondaparinux group and 26 (2%) of 1133 in the enoxaparin group were lost to follow-up at day 49.

F) All kind of outcomes.

**Turpie et al, 2002**

A) Randomisation was done in blocks of four by an independent organisation.

B) Figure 1.

C) Double-blinded.

D) Double-blinded.

E) Figure 1.

F) All kind of outcomes.

**Yokote et al, 2011**

A) Not well reported.

B) Figure 1.

C) Double-blinded.

D) Not reported.

E) Figure 1.

F) All kind of outcomes.
