# Supplementary material for: Efficacy and safety of fondaparinux in elective total hip arthroplasty and hip fracture surgery: a systematic review and meta-analysis
Source: J Orthop Surg Res. 2025 May 29;20:538. doi: 10.1186/s13018-025-05950-6 (PMC12121286; doi:10.1186/s13018-025-05950-6)
Supplement: Supplementary file 3 — Supplementary Material 3 [file 13018_2025_5950_MOESM3_ESM.docx]

**Additional File 1.** Pubmed search strategy.

Search: **fondaparinux AND (orthopedic OR orthopaedic OR "knee arthroplasty" OR "knee replacement" OR "hip arthroplasty" OR "hip replacement")**

("fondaparinux"[MeSH Terms] OR "fondaparinux"[All Fields]) AND ("orthopaedic"[All Fields] OR "orthopedics"[MeSH Terms] OR "orthopedics"[All Fields] OR "orthopedic"[All Fields] OR "orthopaedical"[All Fields] OR "orthopedical"[All Fields] OR "orthopaedics"[All Fields] OR ("orthopaedic"[All Fields] OR "orthopedics"[MeSH Terms] OR "orthopedics"[All Fields] OR "orthopedic"[All Fields] OR "orthopaedical"[All Fields] OR "orthopedical"[All Fields] OR "orthopaedics"[All Fields]) OR "knee arthroplasty"[All Fields] OR "knee replacement"[All Fields] OR "hip arthroplasty"[All Fields] OR "hip replacement"[All Fields])

**Translations**

**fondaparinux:** "fondaparinux"[MeSH Terms] OR "fondaparinux"[All Fields]

**orthopedic:** "orthopaedic"[All Fields] OR "orthopedics"[MeSH Terms] OR "orthopedics"[All Fields] OR "orthopedic"[All Fields] OR "orthopaedical"[All Fields] OR "orthopedical"[All Fields] OR "orthopaedics"[All Fields]

**orthopaedic:** "orthopaedic"[All Fields] OR "orthopedics"[MeSH Terms] OR "orthopedics"[All Fields] OR "orthopedic"[All Fields] OR "orthopaedical"[All Fields] OR "orthopedical"[All Fields] OR "orthopaedics"[All Fields]
