# Supplementary material for: Efficacy and safety of fondaparinux in elective total hip arthroplasty and hip fracture surgery: a systematic review and meta-analysis
Source: J Orthop Surg Res. 2025 May 29;20:538. doi: 10.1186/s13018-025-05950-6 (PMC12121286; doi:10.1186/s13018-025-05950-6)

**Additional File 3.** Publication bias assessed by funnel plots.

**VTE**


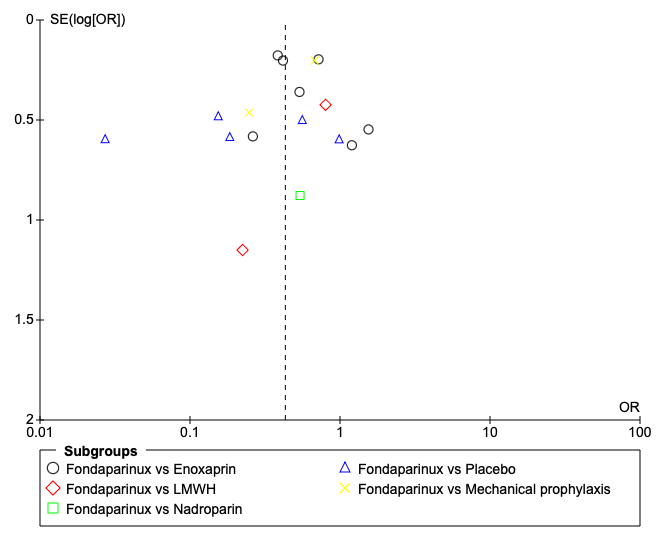


**Distal DVT**


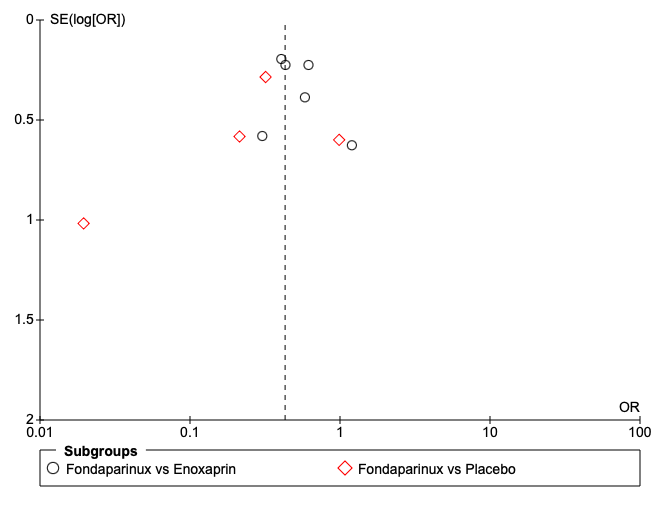


**Proximal DVT**


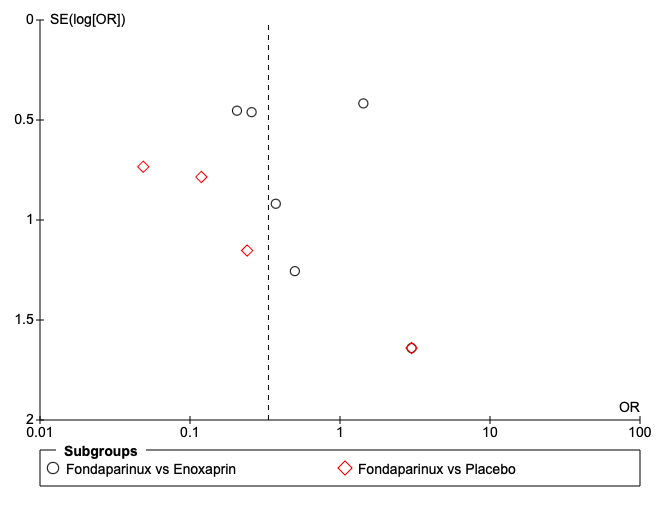


**Non-Fatal PE**


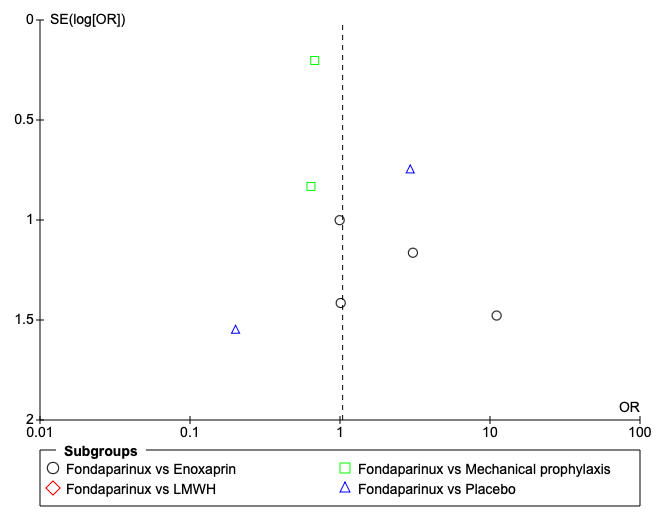


**Mortality rate**


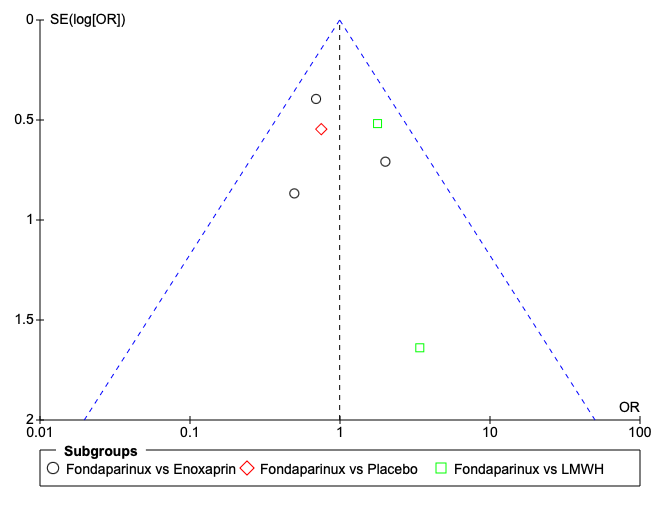


**Minor bleeding**


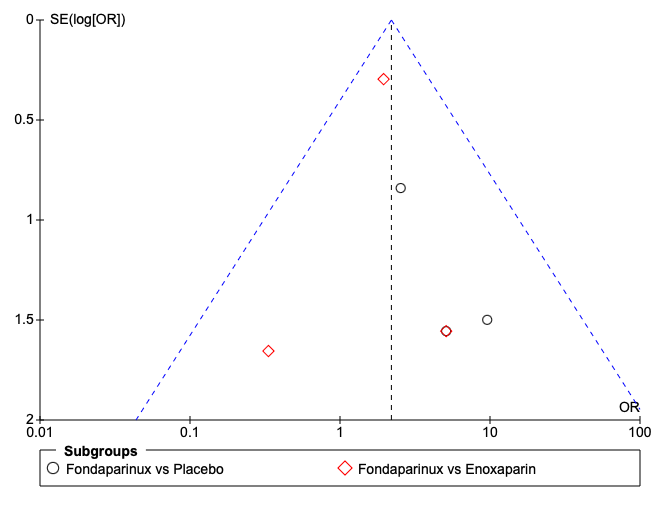


**n Transfusions**


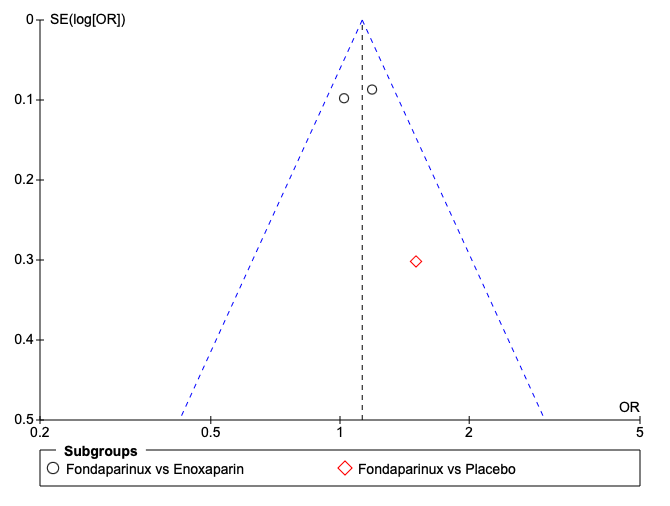

Supplement: Supplementary file 4 — Supplementary Material 4 [file 13018_2025_5950_MOESM4_ESM.docx]
