# Supplementary material for: Efficacy and safety of fondaparinux in elective total hip arthroplasty and hip fracture surgery: a systematic review and meta-analysis
Source: J Orthop Surg Res. 2025 May 29;20:538. doi: 10.1186/s13018-025-05950-6 (PMC12121286; doi:10.1186/s13018-025-05950-6)
Supplement: Supplementary file 5 — Supplementary Material 5 [file 13018_2025_5950_MOESM5_ESM.docx]

**Supplementary Table 2.** Type of examination and time of evaluation.

| **Study** | **Type of examination** | **Time of evaluation** |
| --- | --- | --- |
|  |  |  |
| Eriksson et al. 2001 [20] | Venography | Days 5-11 |
| Eriksson et al. 2003 [19] | Venography | Days 25-32 |
| Fuji et al. 2007 [24] | Venography | Days 11-17 |
| Gao et al. 2024 [26] | Ultrasound | NR |
| Haibier et al. 2023 [10] | Ultrasound | 1 month or when it was suspected. |
| Kawaji et al. 2011 [25] | Ultrasound | Days 3-4 and day 14 |
| Lassen et al. 2002 [12] | Venography | Days 5-11 |
| Migita et al. 2014 [23] | Ultrasound | Day 10 or earlier |
| Okada et al. 2015 [29] | MDCT | Day 7 |
| Sasaki et al. 2011 [9] | Ultrasound | Day 7 |
| Tsuda et al. 2014 [28] | NR | NR |
| Turipie et al. 2002 [21] | Venography | 5-11 days |
| Wang et al. 2024 [11] | NR | NR |
| Yokote et al. 2011 [22] | Ultrasound | Day 11 |
| Yukizawa et al. 2011 [27] | Ultrasound/ Venography with MDCT | Day 7/Day 28 |

NR; Not reported; MDCT; Multidetector row computed tomography.
