# Supplementary material for: Efficacy and safety of fondaparinux in elective total hip arthroplasty and hip fracture surgery: a systematic review and meta-analysis
Source: J Orthop Surg Res. 2025 May 29;20:538. doi: 10.1186/s13018-025-05950-6 (PMC12121286; doi:10.1186/s13018-025-05950-6)
Supplement: Supplementary file 6 — Supplementary Material 6 [file 13018_2025_5950_MOESM6_ESM.docx]

| **Studies** | **Clearly stated aim** | **Consecutive patients** | **Prospective collection data** | **Endpoints** | **Assessment endpoint** | **Follow-up period** | **Loss less than 5%** | **Study size** | **Adequate control group** | **Contemporary group** | **Baseline control** | **Statistical analyses** | **MINORS** |
| --- | --- | --- | --- | --- | --- | --- | --- | --- | --- | --- | --- | --- | --- |
| **Gao et al., 2024 [26]** | 2 | 2 | 0 | 2 | 2 | 2 | 1 | 2 | 2 | 2 | 2 | 2 | 21 |
| **Haibier et al, 2023 [10]** | 2 | 2 | 2 | 2 | 2 | 2 | 2 | 2 | 2 | 2 | 2 | 2 | 24 |
| **Kawaji et al, 2011 [25]** | 2 | 2 | 0 | 2 | 1 | 2 | 0 | 2 | 2 | 2 | 2 | 2 | 19 |
| **Migita et al, 2014 [23]** | 2 | 2 | 2 | 2 | 1 | 2 | 1 | 2 | 1 | 2 | 1 | 2 | 20 |
| **Sasaki et al, 2011 [9]** | 2 | 2 | 2 | 2 | 2 | 2 | 2 | 2 | 2 | 2 | 2 | 2 | 24 |
| **Tsuda et al, 2014 [28]** | 2 | 2 | 2 | 1 | 1 | 2 | 2 | 2 | 2 | 2 | 2 | 2 | 22 |
| **Wang et al, 2024 [11]** | 2 | 2 | 2 | 2 | 2 | 2 | 2 | 2 | 2 | 2 | 2 | 2 | 24 |
| **Yokote et al, 2011 [22]** | 2 | 2 | 2 | 1 | 1 | 1 | 2 | 2 | 2 | 2 | 2 | 2 | 21 |
| **Yukizawa et al, 2012 [27]** | 2 | 2 | 0 | 1 | 1 | 2 | 2 | 2 | 2 | 2 | 2 | 2 | 20 |

**Additional Table 2.** Assessment of the quality of studies through Methodological Index for Non-randomized studies (MINORS).
